# Prediction potential of candidate biomarker sets identified and validated on gene expression data from multiple datasets

**DOI:** 10.1186/1471-2105-8-415

**Published:** 2007-10-26

**Authors:** Michael Gormley, William Dampier, Adam Ertel, Bilge Karacali, Aydin Tozeren

**Affiliations:** 1 School of Biomedical Engineering, Drexel University, Philadelphia, PA, USA

## Abstract

**Background:**

Independently derived expression profiles of the same biological condition often have few genes in common. In this study, we created populations of expression profiles from publicly available microarray datasets of cancer (breast, lymphoma and renal) samples linked to clinical information with an iterative machine learning algorithm. ROC curves were used to assess the prediction error of each profile for classification. We compared the prediction error of profiles correlated with molecular phenotype against profiles correlated with relapse-free status. Prediction error of profiles identified with supervised univariate feature selection algorithms were compared to profiles selected randomly from a) all genes on the microarray platform and b) a list of known disease-related genes (a priori selection). We also determined the relevance of expression profiles on test arrays from independent datasets, measured on either the same or different microarray platforms.

**Results:**

Highly discriminative expression profiles were produced on both simulated gene expression data and expression data from breast cancer and lymphoma datasets on the basis of ER and BCL-6 expression, respectively. Use of relapse-free status to identify profiles for prognosis prediction resulted in poorly discriminative decision rules. Supervised feature selection resulted in more accurate classifications than random or a priori selection, however, the difference in prediction error decreased as the number of features increased. These results held when decision rules were applied across-datasets to samples profiled on the same microarray platform.

**Conclusion:**

Our results show that many gene sets predict molecular phenotypes accurately. Given this, expression profiles identified using different training datasets should be expected to show little agreement. In addition, we demonstrate the difficulty in predicting relapse directly from microarray data using supervised machine learning approaches. These findings are relevant to the use of molecular profiling for the identification of candidate biomarker panels.

## Background

Clinically validated biomarkers are highly valued in cancer pathology for diagnostic and prognostic purposes [1]. Biomarker sets are also used in clinical trials as early indicators of drug efficacy and toxicity [2]. Molecular profiling technologies have the potential to enable high-throughput candidate biomarker identification. Use of oligonucleotide or spotted cDNA microarrays allows for the quantification of the mRNA concentration of thousands of gene products simultaneously [3]. Although measurement of the entire proteome is not yet possible [4], advances in mass spectrometry and chromatography provide similar capabilities at the protein level. Molecular profiling approaches have been applied towards the study of chronic diseases, including muscular dystrophy [5,6], diabetes [7,8], arthritis [9], cardiovascular disease [10] and cancer [11-16]. Microarray studies, in which the class or phenotype (health vs. disease, responders vs. non-responders, etc.) of all samples is known, can be used to identify discriminative features (genes or proteins) that are statistically associated with class distinction [6,9,11,12]. These features can be used as potential biomarker sets to determine the phenotype of new samples and guide therapy appropriately.

Detection of candidate biomarkers from high-dimensional molecular datasets entails separation of signal from noise. As such, techniques adapted from signal processing and machine learning can be applied. The goal of machine learning is to reliably predict the class, or phenotypic state, of a new sample given only a set of measured input variables. The definition of a function that equates input variables to response is called supervised learning. In general, supervised learning consists of three steps: feature selection, decision rule specification and estimation of generalization error [17]. Feature selection is the identification of informative features from noisy or uncorrelated features in the dataset. Decision rule specification involves selection of a classification algorithm and definition of algorithm parameters by cross-validation [14,17,18]. Feature selection and decision rule specification produce a classifier through the use of cross-validation on training data. In this process, there is a risk of overfitting the training data, in which the classifier is trained to recognize noise and not class distinction. The estimation of generalization error, or the misclassification rate expected when the classifier is applied to new samples, can be used to investigate the likelihood of overfitting. An unbiased estimate of the generalization error can only be obtained from independent test data [17].

Feature selection is particularly important in gene expression profiling, in which the number of features (genes) is much larger than the number of observations (microarray data samples). Identification of discriminative features eases the process of data interpretation and communication, decreases computation time for training, and, in biomarker identification, enables the development of reliable clinical assays. Numerous feature selection algorithms can be found in the literature, most of which rank features in a univariate manner, sorting them on the basis of correlation with class distinction [12,18-21]. In molecular profiling studies, univariate methods are used more often than multivariate feature selection methods [22-24] due to their intrinsic simplicity and the higher computational cost of multivariate methods.

Application of supervised feature selection methods in microarray analysis identifies a set of genes whose expression patterns, or profiles, are most correlated with response. However, discriminative feature sets identified in multiple microarray studies of the same disease state or biological condition typically share few common genes [25-27], indicating perhaps that multiple gene subsets can be used as effective biomarker panels in disease classification. Many genes cluster into similar expression profiles and may have similar roles in signalling or metabolic pathways. Variation between studies can also be partially attributed to biological variations between sample populations and technical variations, such as the microarray platform (cDNA vs. oligonucleotide), protocol and analytical techniques used [28,29]. Moreover, selection of discriminative genes within a given dataset is dependent on the selection of training set arrays [30-34].

Given the presence of multiple, generally exclusive expression profiles of disease states such as metastatic breast cancer, it is appropriate to ask whether feature selection identifies expression profiles that classify better than is expected by chance, i.e. better than gene subsets selected randomly. It is also important to determine to what extent technical and biological variability between studies affects the generalization error of classifiers trained on expression profiles. In this study, we analyzed a multitude of publicly available microarray datasets consisting of expression data linked to clinical data for breast cancer, renal cancer, and lymphoma [35-41]. Expression profiles, composed of features associated with response, were created using supervised, univariate feature selection algorithms [12,18]. Our analysis considered multiple microarray technologies (Affymetrix, cDNA spotted arrays, cDNA oligonucleotide arrays), normalization, feature selection and classification methods. Our results point to the efficiency of gene subsets randomly selected from known disease-related genes in the accurate classification of cancer samples according to molecular phenotypes. Results also point to the challenges of predicting relapse directly from microarray data annotated with clinical outcome.

## Results and Discussion

### Simulated datasets confirm the performance of classification algorithms

**Figure 1:**
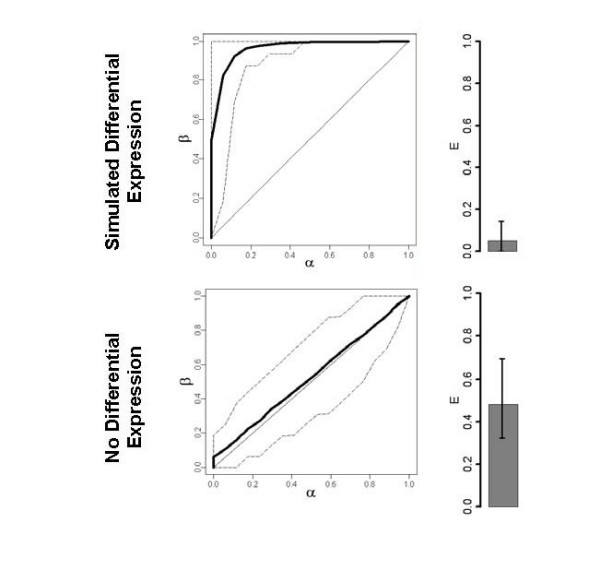
**Classification of Simulated Gene Expression Data**. Receiver operating characteristic (ROC) curves showing classification performance of DLDA classifiers on simulated gene expression data. The symbols α and β are 1-specificity and sensitivity as described in the Methods section. Solid lines are average ROC curves over 100 iterations of training and test set selection. Dashed lines are empirical 95% confidence intervals. Bar plots give the mean 1-AUC (E) with error bars showing empirical 95% CIs.

Analysis of simulated gene expression datasets indicated the effectiveness of the feature selection and classification algorithms used in this study to predict in binary endpoints. Simulated datasets consisting of 100 observations and 1000 features were designed to approximate a binary classification problem [42]. Expression values were drawn from a multivariate normal distribution with mean equal to 0. Differentially expressed genes were simulated from a mixture of the original distribution with a second multivariate normal distribution with mean equal to 2. Our computations, presented in Figure 1, produced highly discriminative decision rules on simulated expression data. Elimination of differential expression, simulated by generating all values from the same distribution, resulted in classifiers with poor classification performance (Figure 1).

### Univariate feature selection is a poor predictor of relapse in breast, lymphoma and renal cancers

**Figure 2:**
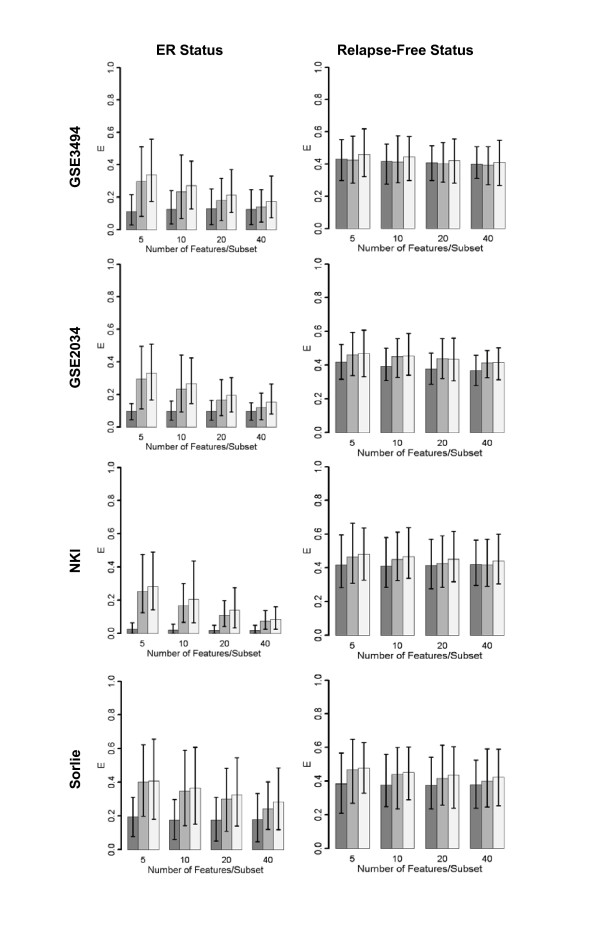
**Prediction error of DLDA classifiers trained and validated on breast cancer datasets**. Column 1: Classifiers trained on ER-status. Column 2: Classifiers trained on relapse-free status. E is the mean 1-AUC of the corresponding set of ROC curves, calculated as described in the Methods section. Error bars show empirical 95% CIs.

**Figure 3:**
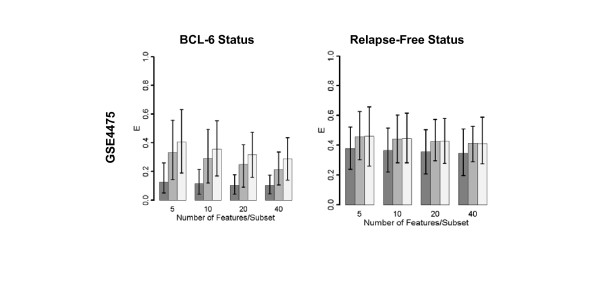
**Prediction error of DLDA classifiers trained and validated on a diffuse large B-cell lymphoma dataset**. Column 1: Classifiers trained on BCL-6 status. Column 2: Classifiers trained on relapse-free status. E is the mean 1-AUC of the corresponding set of ROC curves, calculated as described in the Methods section. Error bars show empirical 95% CIs.

Computations with breast cancer microarray datasets from four independent cohorts of patients (GSE3494, GSE2034, NKI, Sorlie; Table 1) indicate the poor potential of univariate feature selection in predicting relapse-free survival. Figure 2 shows the classification error metric E (described in the methods section) as a function of the number of features used for classification. Columns 1 and 2 in this figure correspond to classification with respect to ER-status and relapse-free survival, respectively. Dark gray bars indicate univariate feature selection whereas light and medium gray bars correspond, respectively, to random selection from either the entire gene set or from an a priori gene set. Error bars indicate the variance over one hundred iterations of the machine learning algorithm. As the figure shows, decision rules trained on relapse-free status classify test samples with low accuracy. Analysis of diffuse large B-cell lymphoma (DLBCL) and conventional renal cell carcinoma (CRCC) datasets similarly yielded high errors in the prediction of relapse-free status (Figure 3, Additional File 1). Survival time is a multi-factorial response variable with many potential confounding factors (ie. lifestyle, age, etc.) that may affect gene expression. The influence of these confounding factors may result in tumor classes that are highly heterogeneous in regards to gene expression. These results indicate the difficulty in predicting relapse-free status in several forms of cancer from microarray data with the use of univariate feature selection.

**Table 1:** Description of microarray datasets

Disease Type	Datasets	Platform	# of Arrays	Restrictions	Reference
Breast Cancer	GSE3494	Affy HG-U133a	251	a	Miller et al. [33]
	GSE2034	Affy HG-U133a	286	b, c	Wang et al. [34]
	NKI	Hu25K	295	d, e, f	Van de Vijver et al. [35]
	Sorlie	cDNA	121	a	Sorlie et al. [36]

Diffuse large B-Cell Lymphoma	Broad	Affy HG-U133a	176	a	Monti et al. [37]
	GSE4475	Affy HG-U133a	220	a	Hummel et al. [38]

Renal Carcinoma	Zhao	cDNA	177	a	Zhao et al. [39]

a. No restrictions

b. Lymph-node negative

c. No adjuvant therapy

d. < 5 cm in diameter

e. < = 52 years at diagnosis

f. No previous history of cancer

### Univariate feature selection as well as randomly chosen features from a priori knowledge set classifies microarray data according to molecular phenotype

In contrast, machine learning methods were able to classify microarray datasets according to molecular phenotype with high accuracy (Figure 2, Additional File 2). In analysis of breast cancer datasets, Figure 2 shows that decision rules trained on ER status classified test samples more accurately than decision rules trained on relapse-free status. These results agree with previous studies in that the expression profiles of many genes seem to be correlated with ER status [11,43]. Estrogen receptor is a hormone-activated transcription factor [44] and also participates in cellular signalling by heterodimerization with membrane-bound receptors such as the endothelial growth factor receptor [44]. Loss of estrogen receptor expression inhibits ER-responsive gene transcription and signaling in downstream pathways and therefore can be expected to affect the expression of downstream genes in a similar manner across tumors. Consistent with the analysis of breast cancer data, lymphoma datasets exhibited low errors in the prediction of BCL-6 status. BCL-6 is a zinc-finger protein that functions as a transcriptional repressor [45] and is expressed in germinal center B cells [46]. In DLBCL, BCL-6 expression, assessed by both immunohistochemistry and RT-PCR, has been associated with better survival in several studies [47,48]. Univariate feature selection may be successful in predicting molecular phenotype due to the fact that expression profiles of many genes are correlated with changes in expression of these transcriptional modulators.

To determine whether gene sets identified with supervised feature selection are uniquely correlated with response, the classification performance of expression profiles was compared with the performance of decision rules created without feature selection (ie. from random gene subsets drawn from either the entire genechip (random selection) or a list of known disease related genes (a priori selection)) (Figure 2). Random selection of subsets of *n *genes gives a baseline error rate expected for classification based on decision rules with *n *features. A priori selection provides a baseline error rate based on the known pathology. In Figure 2, we demonstrate that decision rules that incorporate supervised feature selection classify test samples more accurately than decision rules using a priori selection or random selection. However, in molecular phenotype prediction, the difference in prediction error decreases drastically as the number of features increases. This indicates that the power of univariate supervised feature selection methods lies in identifying small sets of discriminative features. Low prediction error resulting from classification based on multiple, exclusive gene sets has been demonstrated previously by investigating the classification potential of feature sets found down the list of genes ranked by their association with response [32]. Consistent with these previous observations, we demonstrate that randomly selected gene subsets classify molecular phenotype much better than the 50% error rate expected from random classification. In addition, limiting the feature space to genes that have demonstrated disease-relevance in the experimental setting improves classification performance of randomly selected gene sets. These results suggest that the presence of multiple, mostly exclusive biomarker sets identified from different studies [11,35-37] can be partially attributed to the large number of combinations of discriminative feature sets [33].

### Error in predicting relapse is insensitive to normalization and classification algorithms

**Figure 4:**
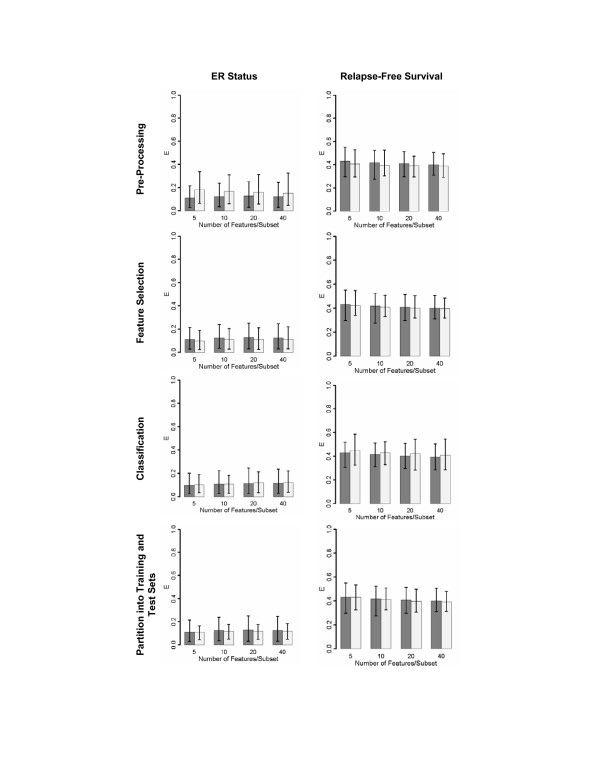
**Senstivity of classifiers to normalization and machine-learning parameters**. Decision rules trained and validated on breast cancer dataset GSE3494 using supervised feature selection. Row 1: Expression values obtained using different pre-processing algorithms. Row 2: Different univariate feature selection methods. Row 3: Different classification schemes. Row 4: Different mode of partition into training and test data. E is the mean 1-AUC for the corresponding set of ROC curves, calculated as described in the Methods section. Error bars are empirical 95% CIs.

Our computations indicate that classification error is only weakly dependent on normalization, feature selection, classification and training/testing partition. Breast cancer dataset GSE3494 was used to assess the effects of these classification parameters on predicted error. Figure 4 shows that these parameters have little effect on prediction for relapse-free survival whereas pre-processing methodologies may have a small impact on the prediction error for ER status.

### Leave-one-out cross-validation scheme may lead to overfitting

**Figure 5:**
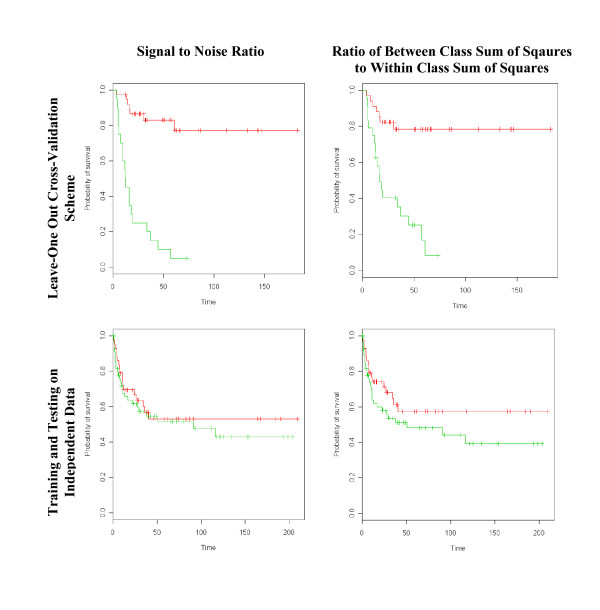
**Kaplan-Meier plots of survival rates for predicted tumor classes with different feature selection/cross-validation methods**. Classifiers trained on the basis of relapse-free status on diffuse large B-cell lymphoma dataset GSE4475. Column 1: Signal to noise ratio. Column 2: Ratio of between class to within class sum of squares. Row 1: Leave-one out cross-validation. All data used for training and testing. Row 2: Training and test sets selected randomly from the dataset. Training based on leave-one out cross-validation.

It has been shown that decision rules based on microarray data are capable of clearly differentiating tumors by outcome when all data is used for feature selection in a leave-one-out cross-validation scheme. Our findings validate previous results in the literature concerning, for example, the prediction of relapse in lymphoma [12]. In their study, Shipp et al. [12] used a machine learning procedure consisting of feature selection with the signal to noise ratio, classifier training by a weighted voting algorithm and leave one out cross-validation on a cohort of 58 lymphoma patients linked to clinical outcome. Importantly, the final geneset was selected from the consensus of all 58 leave-one out models of the data. Using Kaplan Meier analysis [49], Shipp et al. demonstrated a significant difference in survival between the classes predicted by machine learning. We replicated their calculations in this study using a larger microarray dataset (GSE4475, Table 1) and found similar results using both their and our methods of feature selection and classification (Figure 5, Row 1; Additional File 3). Next, we divided the data in GSE4475 into a learning set (randomly selected set of 58 arrays) and test set (remaining 101 arrays) and computed Kaplan Meier survival curves. Results shown in row 2 of Figure 5 (Additional File 3) demonstrate the diminished capacity to identify groups of tumors with different survival rates when complete separation of training and testing sets is maintained in the computations. Thus, when feature selection was included in the cross-validation procedure, such that features selected only from training data were applied to the test data, the difference in survival time between predicted classes decreased. These results suggest the possibility of overfitting in previously reported classifiers based on microarray data linked to clinical outcome.

### Molecular phenotype prediction is maintained in across dataset cross-validation on the same microarray platform

**Figure 6:**
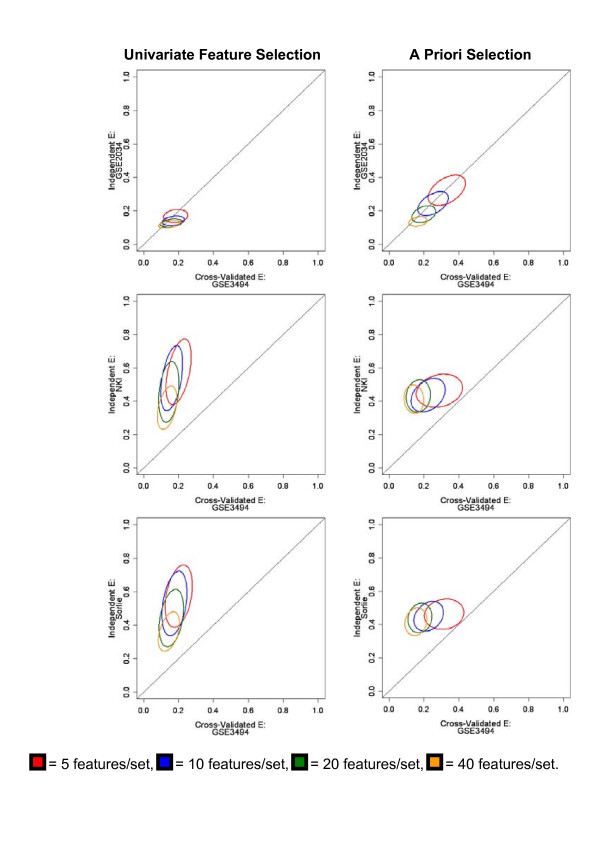
**Prediction error of DLDA classifiers on breast cancer datasets by within-dataset and across-dataset cross-validation**. Decision rules trained on ER-status. Ellipses are centered on the mean 1-AUC of the associated ROC curves. The major axis points in the direction of maximum variance. Lengths of the major and minor axes are proportional to the standard deviation of the data in each direction. Column 1: Prediction error of decision rules based on univariate ranking. Column 2: Prediction error of decision rules based on random selection of features from a subset with a priori disease relevance.

Next, we tested whether prediction error calculated by within dataset cross-validation holds when decision rules trained on one dataset are applied to arrays from other datasets profiling similar populations. Within dataset cross-validation may be biased according to the degree of non-specific correlation between the training and test data. Non-specific correlation can be described as technical noise that arises in sample preparation, hybridization and scanning and results in data collected from the same lab being more highly correlated than data collected in different labs [50]. To investigate this issue further, we used the Affymetrix dataset GSE3494 for developing decision rules for ER status prediction and applied these rules to arrays profiled on either the same (GSE2034) or different microarray platforms (NKI and Sorlie). There was no need to test validation of relapse prediction across datasets since our results showed poor prediction capacity even for within dataset cross-validation. Figure 6 illustrates the results of this analysis in the form of ellipses whose size and shape indicate the distribution of prediction errors. The column on the left (Column 1) belongs to computations using univariate feature selection and the column on the right (Column 2) indicates results corresponding to random selection from an a priori dataset. The figure shows that the prediction error and its variance were much lower on test datasets profiled on the same platform (Figure 6, Row 1) in comparison to test datasets using different platforms (Figure 6, Rows 2 and 3). The same trend held true when the decision rule was based on feature selection from a random set chosen with a priori knowledge (Figure 6, Column 2). These results suggest that decision rules obtained for classification do not accurately predict molecular phenotype in microarray data obtained using different platforms, possibly due to different strategies in probe design, or shortcomings in the matching of probes using probe set annotations [51]. Overall, these results demonstrate that bias resulting from non-specific correlation is negligible when samples are analyzed on the same platform. Results also validate the use of feature selection algorithms to identify small, discriminative feature sets that can be adapted for use in biomarker panels for identifying molecular phenotypes.

## Conclusion

Biomarker sets derived from different global gene expression datasets for the purpose of predicting molecular phenotype or relapse in cancer contain very few common genes [25-27]. In a typical microarray experiment, expression values of many genes are correlated with response [31,33] and therefore, one could predict that multiple biomarker sets could accurately predict the classification of arrays into defined phenotypes. In this study, we used an iterative machine learning approach to determine the prediction potential of biomarker sets chosen using univariate feature selection from training sets selected randomly. On simulated gene expression data, this approach generated several highly discriminative decision rules. Similarly, multiple expression profiles capable of classifying tumors by molecular phenotype were identified in both breast cancer and DLBCL datasets.

We also compared the prediction error resulting from supervised feature selection vs. features selected randomly from either the entire set of genes represented on the microarray or an a priori defined subset of disease-relevant genes. Overall, univariate feature selection led to more accurate classification; however, the difference in prediction errors decreased as the number of features increased. Similar results were also observed in the application of decision rules to samples from other gene expression datasets profiled on the same microarray platform. From this, we conclude that the presence of multiple biomarker sets in the prediction of molecular phenotype arises from the large number of genes correlated with response.

In contrast, decision rules trained on the basis of relapse-free status classified samples with relatively high prediction errors in breast cancer, DLBCL and CRCC datasets. Specifically, prediction error was approximately 40% in all cases that were studied regardless of the method used for feature selection. Overall, these results indicate the difficulty of developing biomarker sets predictive of cancer relapse using a single microarray dataset. Our results do not apply to meta-analytical approaches, in which cancer relapse predictions are obtained by integrating data from multiple microarray datasets prior to machine learning [52-55]. In addition, combined use of clinical information and gene expression data may result in decision rules with better accuracy in predicting relapse [56-58].

## Methods

### Microarray datasets

Publicly available gene expression data for a multitude of cancer types (breast cancer, lymphoma, and renal cancer) were collected from the online repositories Gene Expression Omnibus (GEO) [59] and Stanford Microarray Database (SMD) [60] (Table 1). All datasets used in the study were linked to clinical data including outcome and were further restricted to exclude datasets with less than 100 samples. Expression datasets analyzed in this article included data from multiple platforms (Affymetrix, cDNA, Hu25K), allowing us to assess the platform dependence of our results. Typically, datasets were collected from population-based studies with no age/status restrictions. Two exceptions to this rule are as follows: 1) dataset GSE2034 was restricted to breast cancer patients with lymph-node negative disease and with no adjuvant therapy; and 2) dataset NKI was restricted to patients with tumors less than 5 cm in diameter, and under age 52 at diagnosis (Table 1).

Each microarray dataset was analyzed independently to evaluate the error in predicting relapse (or histological expression of a surrogate biomarker of relapse) using univariate feature selection compared to the resulting error from biomarker sets chosen randomly from either the entire set of genes represented on the microarray (random) or a smaller set of experimentally validated cancer-associated genes (a priori). To this end, we used an iterative supervised, machine-learning approach, described below. For completeness, we tested the dependence of our approach on the use of different pre-processing, feature selection and classification algorithms and cross-validation schemes. The primary focus of our study was on breast cancer, where multiple datasets were available for analysis. Lymphoma and renal carcinoma datasets were used to assess the applicability of our conclusions relevant to breast cancer on other disease states. All work described in this study was carried out using the R statistical environment [61] and was duplicated independently in Matlab unless otherwise noted.

### Pre-processing microarray data

Microarray datasets were collected in raw format when available (GSE3494, GSE4475). Two pre-processing algorithms, Robust Multi-Array Analysis (RMA) [62] and MAS 5.0 [63], were applied to these datasets to determine the effect of pre-processing on downstream analysis. RMA was implemented with the Bioconductor package [64,65] in the R statistical environment [61]. MAS 5.0 was implemented with Array Express Lite. All other datasets were obtained in pre-processed form. The methods used for pre-processing in these cases are summarized briefly as follows. The Broad and GSE2034 datasets were pre-processed using MAS 5.0 [63]. In the GSE2034 dataset, only chips with an average signal intensity of greater than 40 and a background signal of less than 100 were included and probe sets were scaled to a target intensity of 600 [36]. Sorlie and Zhao datasets were obtained from the Stanford Microarray Database (SMD) [60] in log base 2 form. Spots flagged by the scanning software were not included. Missing values were imputed using a nearest neighbour algorithm [66]. Expression values in the NKI dataset were quantified by averaging the intensity across the Cy3 and Cy5 channels and subtracting a local background estimate [37]. Each channel was normalized to the mean intensity across genes.

### Probe set annotation

Affymetrix probe sets were annotated using the hgu133a package [67] in R. Stanford clone IDs were annotated using the SOURCE database [68]. Stanford cDNA datasets consisted of samples processed on different generations of cDNA platforms. To obtain comparable data within each dataset, we limited the dataset to the clone IDs represented on all generations. This step resulted in 8404 and 39414 clone IDs for the Sorlie and Zhao datasets respectively. The NKI probe sets were annotated using Unigene cluster IDs from Unigene build 158 [69]. Retired cluster IDs were identified and re-annotated using records from Unigene. These IDs are sometimes split into multiple clusters. In these cases, annotation was not possible. These probe sets were excluded from the analysis. By retaining only the probe sets that could be definitively annotated, we were left with 8069 probe sets for further analysis.

### Mapping between probe sets and genes

A single probe set representing each gene was selected due to the varying redundancy of gene representation on microarray platforms. In datasets compiled in this study, approximately 60% of genes were represented by a single probe set. Genes represented by multiple probe sets were dealt with in the following manner. For Affymetrix datasets, probe set suffixes were used to remove redundant probe sets. For the HG-U133a chip, probe sets are encoded with _at, _s_at and _x_at suffixes that describe the quality of probe design [70]. All _x_at probe sets (~10% of probe sets on the array) were excluded. If a gene were represented by an _at probe set, and multiple _s_at probe sets, the _at probe set was selected. Approximately 20% of redundant probe sets could be dealt with in this manner. In cases in which a unique probe set could not be chosen by the suffix, the average expression value of the remaining probe sets was used. For the non-Affymetrix probe sets, a unique probe set was chosen by selecting the probe set with the highest variance across samples.

### Feature selection

Microarray datasets were iteratively divided into learning sets (LS) and test sets (TS) to create a population of classifiers and determine their classification performance in a Monte Carlo cross-validation approach [71]. Two types of response variables were used to divide tumors into groups of poor prognosis and good prognosis, either histological expression of biomarkers (ER status in breast cancer, BCL-6 status in lymphoma) or relapse-free survival, in which relapse is defined as disease recurrence or death from disease. Learning sets and test sets were selected by first dividing datasets by response variable and then randomly selecting equal proportions of arrays from each class. Two different partitions were used: 2/3 LS, 1/3 TS and 1/2 LS, 1/2 TS. Learning sets were used to select informative features and train the decision rule. Genes were selected from learning set data in a supervised manner using a univariate feature selection algorithm, implemented with the stat.bwss function in the sma package [72]. Briefly, each gene was ranked by the ratio of between class sum of squares to within class sum of squares. High scoring genes have large between class variances and small within class variances and are therefore correlated with class distinction. A second univariate feature selection method [12] was used to determine if our results were sensitive to feature selection algorithms. In this second method, genes are ranked by the signal to noise ratio, namely the ratio of the difference in class-specific means to the sum of the class-specific standard deviations [12]. This is quite similar to the two-sample t-statistic. We use the term signal to noise ratio to maintain consistency with previous literature in the field. For comparison, genes were selected randomly from either the entire list of genes represented on the array (random), or a list of experimentally validated disease-related genes obtained from the Ingenuity Pathways Database [73] (a priori). All three feature sets (feature selected, a priori, and random) were used in downstream analyses.

### Classification

Two classification algorithms, diagonal linear discriminant analysis (DLDA) and k- nearest neighbour (NN, k = 3), were used to create decision rules on the basis of training data. The NN algorithm classified test samples by the class of the three closest samples in the training set using Euclidean distance [72]. DLDA is based on the maximum likelihood discriminant rule [72]. These relatively simple classifiers have been shown to give accurate classifications when used to analyze expression data and appear to perform as well as or better then more sophisticated algorithms, such as support vector machines, and resampling methods, such as bagging or boosting [72,74].

### Validation

To obtain an estimate of generalization error, decision rules were applied to thecorresponding test sets. The confidence (δ) with which each sample was classified was calculated as follows:

δ=dR(dR+dN)
 MathType@MTEF@5@5@+=feaafiart1ev1aaatCvAUfKttLearuWrP9MDH5MBPbIqV92AaeXatLxBI9gBaebbnrfifHhDYfgasaacH8akY=wiFfYdH8Gipec8Eeeu0xXdbba9frFj0=OqFfea0dXdd9vqai=hGuQ8kuc9pgc9s8qqaq=dirpe0xb9q8qiLsFr0=vr0=vr0dc8meaabaqaciaacaGaaeqabaqabeGadaaakeaaiiGacqWF0oazcqGH9aqpdaWcaaqaaiabdsgaKnaaBaaaleaacqWGsbGuaeqaaaGcbaWaaeWaaeaacqWGKbazdaWgaaWcbaGaemOuaifabeaakiabgUcaRiabdsgaKnaaBaaaleaacqWGobGtaeqaaaGccaGLOaGaayzkaaaaaaaa@39ED@

in which d_R _and d_N _are the distances predicted by the decision rule to the poor and good prognosis classes, respectively. Samples are classified as good prognosis if the score is greater than 0.5 and vice-versa.

With this methodology, the classification performance of the decision rulescould be visualized and compared with the use of receiver operating characteristic (ROC) curves [75]. ROC curves plot sensitivity, or detection rate, (β) against 1-specificity, or false alarm rate (α).

β=TruePositives(TruePositives+FalseNegatives)
 MathType@MTEF@5@5@+=feaafiart1ev1aaatCvAUfKttLearuWrP9MDH5MBPbIqV92AaeXatLxBI9gBaebbnrfifHhDYfgasaacH8akY=wiFfYdH8Gipec8Eeeu0xXdbba9frFj0=OqFfea0dXdd9vqai=hGuQ8kuc9pgc9s8qqaq=dirpe0xb9q8qiLsFr0=vr0=vr0dc8meaabaqaciaacaGaaeqabaqabeGadaaakeaaiiGacqWFYoGycqGH9aqpdaWcaaqaaiabdsfaujabdkhaYjabdwha1jabdwgaLjabdcfaqjabd+gaVjabdohaZjabdMgaPjabdsha0jabdMgaPjabdAha2jabdwgaLjabdohaZbqaaiabcIcaOiabdsfaujabdkhaYjabdwha1jabdwgaLjabdcfaqjabd+gaVjabdohaZjabdMgaPjabdsha0jabdMgaPjabdAha2jabdwgaLjabdohaZjabgUcaRiabdAeagjabdggaHjabdYgaSjabdohaZjabdwgaLjabd6eaojabdwgaLjabdEgaNjabdggaHjabdsha0jabdMgaPjabdAha2jabdwgaLjabdohaZjabcMcaPaaaaaa@6820@

α=FalsePositives(FalsePositives+TrueNegatives)
 MathType@MTEF@5@5@+=feaafiart1ev1aaatCvAUfKttLearuWrP9MDH5MBPbIqV92AaeXatLxBI9gBaebbnrfifHhDYfgasaacH8akY=wiFfYdH8Gipec8Eeeu0xXdbba9frFj0=OqFfea0dXdd9vqai=hGuQ8kuc9pgc9s8qqaq=dirpe0xb9q8qiLsFr0=vr0=vr0dc8meaabaqaciaacaGaaeqabaqabeGadaaakeaaiiGacqWFXoqycqGH9aqpdaWcaaqaaiabdAeagjabdggaHjabdYgaSjabdohaZjabdwgaLjabdcfaqjabd+gaVjabdohaZjabdMgaPjabdsha0jabdMgaPjabdAha2jabdwgaLjabdohaZbqaamaabmaabaGaemOrayKaemyyaeMaemiBaWMaem4CamNaemyzauMaemiuaaLaem4Ba8Maem4CamNaemyAaKMaemiDaqNaemyAaKMaemODayNaemyzauMaem4CamNaey4kaSIaemivaqLaemOCaiNaemyDauNaemyzauMaemOta4KaemyzauMaem4zaCMaemyyaeMaemiDaqNaemyAaKMaemODayNaemyzauMaem4CamhacaGLOaGaayzkaaaaaaaa@6914@

Classification performance was determined from the area under the curve (AUC). Good classifiers have high detection rates across the range of false alarm rates and therefore have a large AUC. ROC curves were generated for each classifier using the ROC package in R [76]. The score δ was divided into thresholds and β and α were calculated at each cut-off point. The AUC of each classifier was then calculated using the sum of trapezoids. The entire process of feature selection, decision rule specification and estimation of generalization error was repeated 100 times to determine the expected performance of each gene set on a randomly selected set of samples. Average ROC curves were calculated from the distribution of detection rates at given false alarm rates [77]. Empirical confidence intervals were obtained as the 97.5% and 2.5% quantiles of this distribution [77]. The expected classification performance was quantified using a prediction error metric E defined as the mean 1-AUC. A smaller E value corresponds to a more accurate classifier.

### Simulated expression data

Simulated microarray datasets were generated to verify that the machine learning algorithm described above leads to accurate classification. Simulated expression data was created in the manner described by Bura and Pfeiffer [42]. Datasets consisted of 100 observations with 1000 variables each, corresponding to arrays and genes respectively. Half of the observations were labelled as class 1 and the remainder were labelled class 0. All data for class 0 samples were drawn from a multivariate normal distribution with mean 0 and a covariance matrix of Σ. Five percent of genes were simulated to be differentially expressed. For class 1 samples, differentially expressed genes were drawn from a mixture of two multivariate normal distributions with means 0 and 2 and covariance structure Σ. The mixing probability was 1/2. Non-differentially expressed genes were generated from the same distribution as class 0 samples. The covariance matrix Σ = σ_ij _was generated with a block structure with σ_ij _= 0.2 for | j-i | ≤ 5 and 0 otherwise.

### Statistical significance of molecular profile prediction from microarray data

To determine the significance of the calculated prediction error metric E for molecular profile prediction in breast cancer and lymphoma, the machine learning algorithm was repeated 1000 times with permuted class labels. An empirical p-value was calculated as the fraction of decision rules based on permuted class labels that performed better than the expected classification performance, E (described above), of decision rules based on true class labels. Permutation processes give an estimate of the likelihood that the true E value could be obtained by chance alone and are frequently used in similar studies for this purpose [21,78]. Results are summarized in Additional File 2.

### Independent validation

In addition to cross-validated generalization error, we determined the classification accuracy across datasets. To this end, decision rules trained on one dataset were tested in both the corresponding test subset and datasets obtained by other laboratories, resulting in E values for each test case. For across platform comparisons (Affymetrix vs. Hu25K, Affymetrix vs. cDNA), probe sets were matched by annotation to Entrez Gene identifiers.

## List of abbreviations

ROC, Receiver Operating Characteristic; ER, Estrogen Receptor; BCL-6, B-cell CLL/lymphoma 6; cDNA, Complimentary Deoxyribonucleic Acid; mRNA, Messenger Ribonucleic Acid; DLBCL, Diffuse Large B-cell Lymphoma; CRCC, Conventional Renal Cell Carcinoma; RT-PCR, Reverse Transcription Polymerase Chain Reaction; GEO, Gene Expression Omnibus; SMD, Stanford Microarray Database; RMA, Robust Multi-Array Analysis; LS, Learning Set; TS, Test Set; DLDA, Diagonal Linear Discriminant Analysis; NN, Nearest Neighbor; AUC, Area Under the Curve;

## Authors' contributions

MG and AT conceived and developed the research plan. MG implemented the integration of algorithms, performed the computations and wrote the manuscript draft. WD, AE and BK also contributed to the conception of the study and implementation of the strategy. All authors read and approved the final manuscript.

## Supplementary Material

Additional file 1 **Prediction error of DLDA classifiers on lymphoma (Broad) and renal carcinoma (Zhao) datasets**. Classifiers trained to predict relapse-free status. E is the mean 1-AUC of the corresponding set of ROC curves, calculated as described in the Methods section. Error bars show empirical 95% CIs. Click here for file

Additional file 2 **Significance of prediction error (P values) of DLDA classifiers trained to predict molecular phenotype**. Bold entries indicate significant P-values < = 0.01. Click here for file

Additional file 3 **Kaplan-Meier plots of survival rates for tumor classes with different classification/cross-validation methods**. Classifiers trained on the basis of relapse-free status on diffuse large B-cell lymphoma dataset GSE4475. Column 1: Weighted-voting algorithm. Column 2: DLDA. Row 1: Leave-one out cross-validation. All data used for training and testing. Row 2: Training and test sets selected randomly from the dataset. Training based on leave-one out cross-validation. Click here for file
