# Supplementary material for: Prediction potential of candidate biomarker sets identified and validated on gene expression data from multiple datasets
Source: BMC Bioinformatics. 2007 Oct 26;8:415. doi: 10.1186/1471-2105-8-415 (PMC2211325; doi:10.1186/1471-2105-8-415)
Supplement: Additional file 1 — Prediction error of DLDA classifiers on lymphoma (Broad) and renal carcinoma (Zhao) datasets. Classifiers trained to predict relapse-free status. E is the mean 1-AUC of the corresponding set of ROC curves, calculated as described in the Methods section. Error bars show empirical 95% CIs. [file 1471-2105-8-415-S1.doc]

|  | **Relapse-Free Status** |
| --- | --- |
| **Broad** | **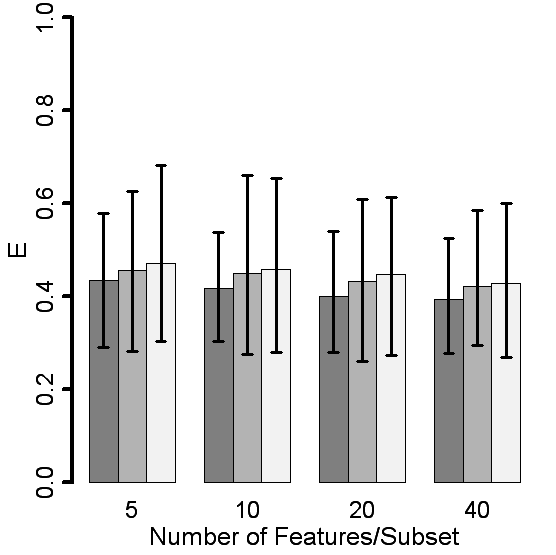** |
| **Zhao** | **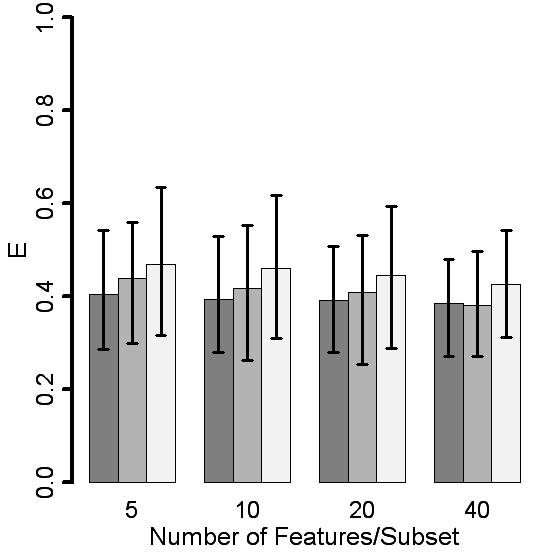** |

= supervised feature selection, = a priori feature selection, = random feature selection.
