# Supplementary material for: Prediction potential of candidate biomarker sets identified and validated on gene expression data from multiple datasets
Source: BMC Bioinformatics. 2007 Oct 26;8:415. doi: 10.1186/1471-2105-8-415 (PMC2211325; doi:10.1186/1471-2105-8-415)
Supplement: Additional file 2 — Significance of prediction error (P values) of DLDA classifiers trained to predict molecular phenotype. Bold entries indicate significant P-values < = 0.01. [file 1471-2105-8-415-S2.doc]

| Disease Type | Dataset | Feature Selection | Number of Features | | | |
| --- | --- | --- | --- | --- | --- | --- |
|  |  |  | 5 | 10 | 20 | 40 |
| Breast Cancer | GSE3494 | Univariate | **0.001** | **0.004** | **0.007** | **0.005** |
|  |  | A Priori | 0.181 | 0.087 | 0.044 | 0.017 |
|  |  | Random | 0.119 | 0.058 | 0.017 | **0.008** |
|  | GSE2034 | Univariate | **< 0.001** | **< 0.001** | **0.001** | **0.002** |
|  |  | A Priori | 0.134 | 0.059 | 0.012 | **0.001** |
|  |  | Random | 0.079 | 0.025 | **0.008** | **0.002** |
|  | NKI | Univariate | **< 0.001** | **< 0.001** | **< 0.001** | **< 0.001** |
|  |  | A Priori | 0.154 | 0.052 | **0.009** | **< 0.001** |
|  |  | Random | 0.054 | 0.016 | 0.011 | **0.002** |
|  | Sorlie | Univariate | **0.010** | **0.009** | **0.010** | 0.012 |
|  |  | A Priori | 0.256 | 0.151 | 0.093 | 0.027 |
|  |  | Random | 0.0256 | 0.158 | 0.097 | 0.065 |
| Diffuse  large B-cell Lymphoma | GSE4475 | Univariate | **0.002** | **0.002** | **0.003** | **0.001** |
|  |  | A Priori | 0.197 | 0.138 | 0.075 | 0.030 |
|  |  | Random | 0.230 | 0.119 | 0.081 | 0.052 |
