# Supplementary material for: Prediction potential of candidate biomarker sets identified and validated on gene expression data from multiple datasets
Source: BMC Bioinformatics. 2007 Oct 26;8:415. doi: 10.1186/1471-2105-8-415 (PMC2211325; doi:10.1186/1471-2105-8-415)
Supplement: Additional file 3 — Kaplan-Meier plots of survival rates for tumor classes with different classification/cross-validation methods. Classifiers trained on the basis of relapse-free status on diffuse large B-cell lymphoma dataset GSE4475. Column 1: Weighted-voting algorithm. Column 2: DLDA. Row 1: Leave-one out cross-validation. All data used for training and testing. Row 2: Training and test sets selected randomly from the dataset. Training based on leave-one out cross-validation. [file 1471-2105-8-415-S3.doc]

|  | **Weighted Voting Classifier** | **Diagonal Linear Discriminant Analysis** |
| --- | --- | --- |
| **Training and Testing on Same Data** | **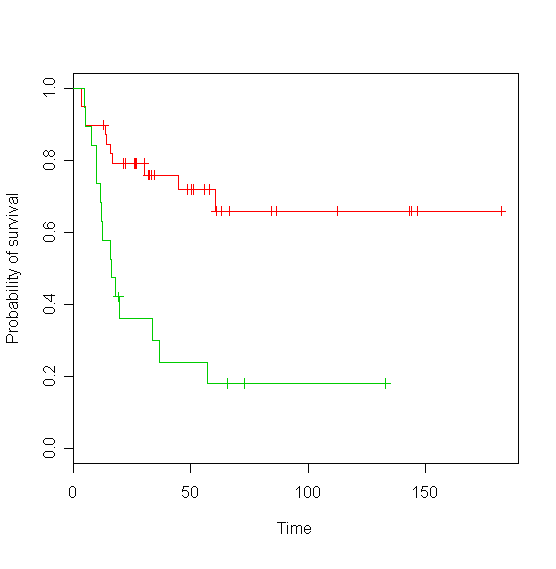** | **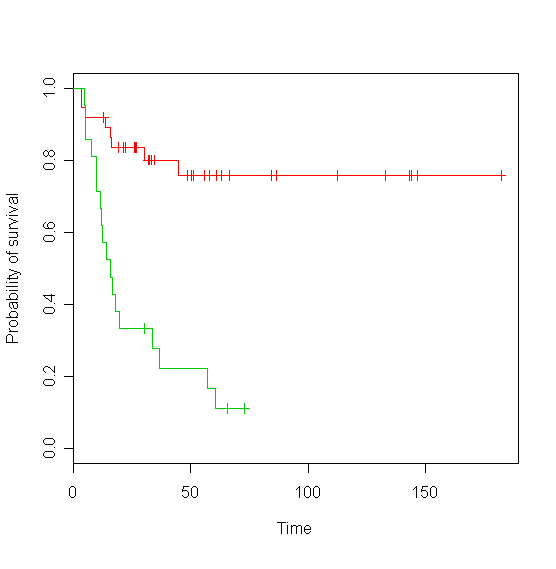** |
| **Training and Testing on Independent Data** | **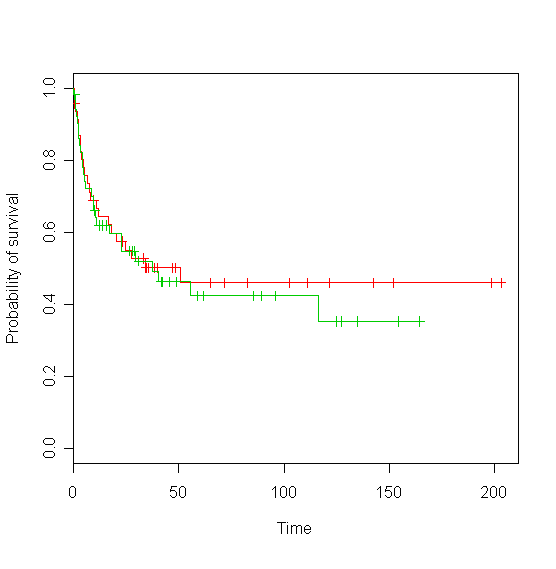** | **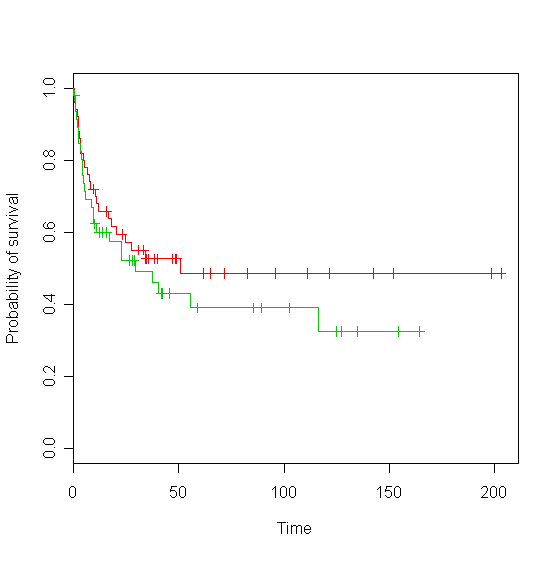** |
